# Trafficking of Annexins during Membrane Repair in Human Skeletal Muscle Cells

**DOI:** 10.3390/membranes12020153

**Published:** 2022-01-26

**Authors:** Coralie Croissant, Céline Gounou, Flora Bouvet, Sisareuth Tan, Anthony Bouter

**Affiliations:** Institute of Chemistry and Biology of Membranes and Nano-Objects, UMR 5248, CNRS, University of Bordeaux, IPB, F-33600 Pessac, France; coralie33400@free.fr (C.C.); celine.gounou@u-bordeaux.fr (C.G.); flora.bouvet@u-bordeaux.fr (F.B.); sisareuth.tan@u-bordeaux.fr (S.T.)

**Keywords:** annexin, membrane repair, skeletal muscle, correlative light and electron microscopy

## Abstract

Defects in membrane repair contribute to the development of muscular dystrophies, such as Miyoshi muscular dystrophy 1, limb girdle muscular dystrophy (LGMD), type R2 or R12. Deciphering membrane repair dysfunctions in the development of muscular dystrophies requires precise and detailed knowledge of the membrane repair machinery in healthy human skeletal muscle cells. Using correlative light and electron microscopy (CLEM), we studied the trafficking of four members of the annexin (ANX) family, in myotubes damaged by laser ablation. Our data support a model in which ANXA4 and ANXA6 are recruited to the disruption site by propagating as a wave-like motion along the sarcolemma. They may act in membrane resealing by proceeding to sarcolemma remodeling. On the other hand, ANXA1 and A2 exhibit a progressive cytoplasmic recruitment, likely by interacting with intracellular vesicles, in order to form the lipid patch required for membrane resealing. Once the sarcolemma has been resealed, ANXA1 is released from the site of the membrane injury and returns to the cytosol, while ANXA2 remains accumulated close to the wounding site on the cytoplasmic side. On the other side of the repaired sarcolemma are ANXA4 and ANXA6 that face the extracellular milieu, where they are concentrated in a dense structure, the cap subdomain. The proposed model provides a basis for the identification of cellular dysregulations in the membrane repair of dystrophic human muscle cells.

## 1. Introduction

Cells from tissues, such as skeletal or cardiac muscle, gut epithelium or vascular endothelium, are exposed to mechanical stress, which often induces the formation of tears in their plasma membrane [1]. Normal cells are able to repair these ruptures at the minute scale, through a coordinated Ca^2+^-dependent response, including intracellular vesicle recruitment and cell membrane remodeling, driven by a protein machinery that involves dysferlin, MG-53, AHNAK and ANX [2,3]. ANX (12 members in humans) are proteins that share the property of binding to negatively charged lipid membranes, principally those containing phosphatidylserine, in a Ca^2+^-dependent manner [4]. ANX present a common C-terminal membrane-binding core, which has the shape of a slightly curved rhomboid, the convex face containing the Ca^2+^-binding sites responsible for membrane interaction. The N-terminal end, variable in length and in sequence, protrudes from the concave side and faces the cytosol. It contains phosphorylation sites and binding sites for various molecular partners and is assumed to be responsible for the functional specificity of ANX [5]. By rapidly (second scale) translocating to the disruption site, many ANX (A1 to A7) have been shown to participate in membrane resealing in various tissues and cell types, such as epithelial cells [6], cancer cells [6,7,8,9], endothelial cells [10], trophoblasts [11], pericytes [12] or skeletal myocytes [13,14,15,16,17,18].

Defective membrane repair leads to cell death and may contribute to the development of degenerative diseases, such as muscular dystrophies [3,19,20]. Major attention has therefore focused on membrane repair in skeletal muscle cells. In this context, LGMD type R2 (formerly 2B) is the most studied and best documented pathology. LGMDR2 is due to mutations in the *dysferlin* gene, which prevent membrane resealing and lead to skeletal muscle degeneration [19]. Muscle damage and disease severity in LGMDR2 patients correlate with an increased expression of ANXA2, which is likely an attempt for the cell to tackle the defect of the membrane repair [21]. The progressive release of ANXA2, from the unrepaired damaged myofibers into the extracellular milieu, causes accumulation of fibro-adipogenic progenitors (FAP) and macrophages [22,23]. Finally, ANXA2 induces the differentiation of FAP into adipocytes, which leads to the adipogenic replacement of myofibers and, consequently, muscle loss [22]. These observations stress the importance of better characterizing functions of ANX in membrane repair, in both physiological and pathophysiological conditions. Except for ANXA5 [15] and ANXA6 [17], most previous studies have been performed in animal models [13,14,16,18].

Here, therefore, we focused on the analysis of ANXA1-A4 in the membrane repair of human skeletal muscle cells. We first studied their expression in myoblasts and myotubes and observed the absence of ANXA3. We then compared the kinetics of recruitment of ANX to the site of the membrane injury after laser ablation. To complete the analysis, CLEM experiments were performed in order to determine, by transmission electron microscopy (TEM), the accurate localization of ANX at the wounding site. Our study provides novel information on the trafficking of ANX in human skeletal muscle cells submitted to membrane damage. A model of sarcolemma repair involving ANX is proposed.

## 2. Materials and Methods

### 2.1. Culture of Human Skeletal Muscle Cells

The healthy LHCN-M2 (referred to hereafter as LHCN) cell line, which was established from satellite cells of the pectoralis major muscle of a 41-year-old subject [24] and provided by the platform for immortalization of human cells from the Center of Research in Myology (Paris, France), was cultured as previously described [17].

### 2.2. Western Blot

Preparation of protein extracts and western blot analysis were performed as previously described [17]. ANXA1 (37 kDa), ANXA2 (37 kDa), and ANXA4 (35 kDa) were respectively detected with rabbit polyclonal anti-ANXA1 (PA1006, Boster, Pleasanton, CA, USA), mouse monoclonal anti-ANXA2 (WH0000302M1, Sigma, Saint-Louis, MI, USA) and rabbit polyclonal anti-ANXA4 (PA1007-1, Boster, Pleasanton, CA, USA). Attempts to detect ANXA3 were performed using mouse monoclonal anti-ANXA3 (sc-390502, Santa Cruz Biotechnology, Dallas, TX, USA) or rabbit polyclonal anti-ANXA3 (PA1510, Boster, Pleasanton, CA, USA). GAPDH (loading control) was detected with a rabbit anti-GAPDH polyclonal antibody (Santa Cruz Biotechnology, Dallas, TX, USA). At least three independent experiments were performed for each ANX. Quantification of relative intensity of protein band was performed by ImageJ software. A Wilcoxon test was performed to identify putative statistical difference (*p* < 0.05) between values obtained for myoblasts and myotubes. These results are presented in Figure S1B.

### 2.3. Immunocytofluorescence

Cells were immunostained as previously described [17]. Primary antibodies were those used in western blot. Secondary antibodies were Alexa Fluor 488-coupled anti-mouse goat antibody or Alexa Fluor 488-coupled anti-rabbit goat antibody (Thermo Fisher Scientific, Waltham, MA, USA).

### 2.4. Subcellular Trafficking of ANX Fused to Fluorescent Proteins in Damaged Myotubes

The pA1-GFP and pA2-GFP plasmids, which were constructed by cloning, respectively, *ANXA1* and *ANXA2* cDNA into the pEGFP-N3 (Takara Bio USA, Mountain View, CA, USA) plasmid, and the pA6-GFP plasmid, which was constructed by cloning *ANXA6* cDNA into the pEGFP-N1 (Takara Bio USA, Mountain View, CA, USA) plasmid, were a gift from Prof. Volker Gerke (University of Muenster, Münster, Germany). The pA4-GFP was constructed by replacing the *ANXA1* by *ANXA4* cDNA, which was amplified from the pDONR-A4 vector (Proteogenix, Schiltigheim, France) and digested by *Not*I and *Sma*I, by using the In-Fusion^®^ HD cloning kit (Takara Bio USA, Mountain View, CA, USA). The pA1-mCherry and pA4-mCherry plasmids were constructed by replacing the *GFP* by *mCherry* cDNA into respectively pA1-GFP and pA4-GFP plasmids by using the In-Fusion^®^ HD cloning kit (Takara Bio USA, Mountain View, CA, USA). The *mCherry* cDNA was amplified from the pA2-mCherry plasmid (a gift from Prof Volker Gerke, University of Muenster, Germany) and digested by *Not*I and *Sma*I.

LHCN myoblasts at 65 h of differentiation were transfected as previously described [17]. For myotubes co-expressing ANX-GFP together with ANX-mCherry, fluorescent signals of GFP and mCherry are respectively presented in green and magenta within the manuscript, for a better visualization. Magenta is indeed the color that the human eye best distinguishes from green [25].

Membrane damage was performed by laser ablation as previously described [17,26]. Briefly, myotubes were formed and cultured in a 35-mm glass bottom dish equipped with a square-patterned coverslip (MatTek, Ashland, MA, USA). To induce membrane damage, cells were irradiated at 820 nm with a tunable pulsed depletion laser Mai Tai HP (Spectra-Physics, Irvine, USA) of an upright two-photon confocal scanning microscope (TCS SP5, Leica, Wetzlar, Germany) equipped with an HCX APO L U-V-I 63.0 × 0.90 water lens. Irradiation consisted of 1 scan (1.3 s) of a 1 µm × 1 µm area with a power of 110 (±5) mW. Images of 512 × 512 were acquired at 1.3 s intervals with pinhole set to 1 Airy unit.

At least three independent experiments were performed for each ANX-GFP, either alone or paired to ANX-mCherry. Each experiment included the analysis of at least five damaged myotubes. All cellular events that are described in this article, such as wave-like propagation, accumulation into the cap subdomain or difference in kinetics of recruitment between ANX, were observed in at least 75% of damaged myotubes.

### 2.5. Immuno-TEM

Analysis of the subcellular localization of ANX-GFP in damaged cells by CLEM was performed as previously described [17]. Briefly, laser-damaged myotubes were fixed in 1% glutaraldehyde solution for 20 min at room temperature. They were subsequently incubated in 25 mM ammonium chloride for 15 min and permeabilization and saturation were performed in a mixture of 2% BSA and 0.1% Triton X-100 in D-PBS for 10 min. Primary antibody at 1:100 and secondary Alexa Fluor 488- and gold nanoparticles-conjugated anti-mouse goat antibody (FluoroNanogold, Nanoprobes, Yaphank, NY, USA) at 1:100 in 2% BSA solution, were successively incubated with myotubes for 1 h at 37 °C. After three rinses, myotubes were observed by fluorescence microscopy. Cells were then post-fixed overnight at 4 °C in a mixture of 4% paraformaldehyde and 2% glutaraldehyde in 0.1 M cacodylate buffer (pH 7.4). Signal amplification was performed using the HQ silver kit (Nanoprobes, Yaphank, NY, USA) according to the manufacturer’s instructions. Cells were treated with 1% osmium tetroxide in 0.1 M cacodylate buffer for 1 h at room temperature and then dehydrated with ethanol and finally embedded in Epon-Araldite. Thin sections (65 nm) were collected using EM UC7 ultramicrotome (Leica, Wetzlar, Germany) and stained successively with 5% uranyl acetate and 1% lead citrate. TEM observation was performed with a FEI CM120 operated at 120 kV. Images were recorded with a USC1000 slow scan CCD camera (Gatan, Pleasanton, CA, USA).

## 3. Results

### 3.1. Expression of ANX in Human Skeletal Muscle Cells

**Figure 1:**
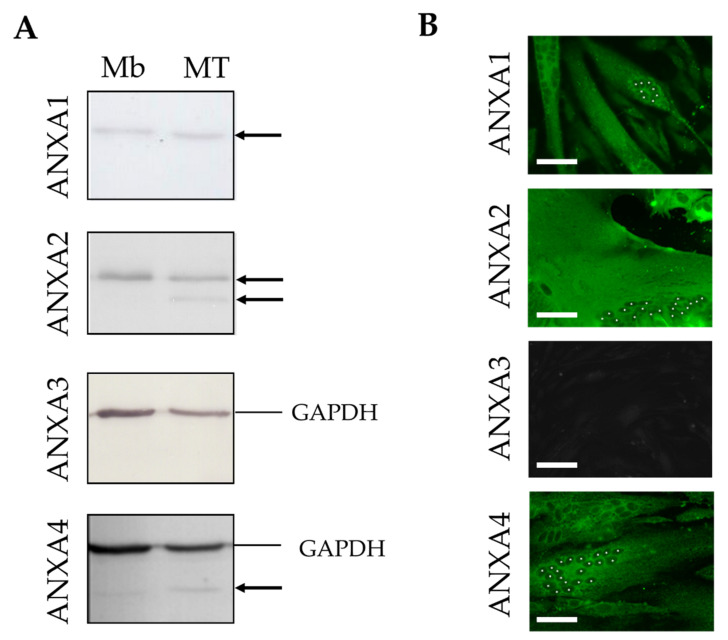
Expression and subcellular distribution of ANX in human myoblasts and myotubes. (**A**) Cellular content in endogenous ANXA1 to A4 in LHCN myoblasts (Mb) and myotubes (MT) was quantified through western blot analysis. Black arrows indicate bands corresponding to ANX. The four western blot experiments were performed simultaneously with the same protein extracts (10 µg). The experimental series was repeated at least three times with independent samples. GAPDH, used as a loading control, was immunodetected together with ANXA3 and ANXA4, but not ANXA1 and ANXA2 that exhibit a similar molecular weight (37 kDa). The whole membranes, as well as quantitative analysis, are presented in Figure S1. (**B**) Subcellular localization of endogenous ANXA1 to A4 (green) in LHCN myotubes by immunocytofluorescence. White asterisks indicate nuclei in one myotube per image. Results in LHCN myoblasts are presented in Figure S2. Scale bars: 50 µm.

The presence of ANXA1 to A4 in human skeletal muscle cells was analyzed by western blot (Figure 1A and Figure S1) and immunocytofluorescence (Figure 1B and Figure S2) from the healthy human myogenic cell line LHCN, either at myoblast stage or differentiated into myotubes. ANXA2, which was detected by a monoclonal antibody, displayed a strong signal, suggesting a significant expression within human skeletal muscle cells (Figure 1A). We noted the presence of a specific short isoform of ANXA2 in myotubes compared to myoblasts (Figure 1A). This isoform may result from a partial proteolysis of ANXA2 or post-translational modification. It has indeed been observed that the N-terminal part of ANXA2 could be cleaved in human smooth muscle cells or fibroblasts [27,28]. In addition, the N-terminal moiety is also subject to post-translational modifications, which are crucial for interaction with S100A10 and the formation of the heterotetramer [29,30]. ANXA1 and ANXA4 were similarly expressed in myoblasts and myotubes (Figure 1A). Both were weakly detected, despite the use of a polyclonal antibody, which suggested they were sparsely expressed. Finally, ANXA3 was undetectable, whatever the technique used (Figure 1A,B), either with mouse monoclonal (not shown) or rabbit polyclonal antibodies (Figure 1A,B), suggesting it was not expressed in human skeletal muscle.

We then focused on the subcellular localization of ANX in LHCN myoblasts and myotubes by immunocytofluorescence (Figure 1B and Figure S2). We observed that ANXA1 was localized both in the nucleus and cytoplasm of myoblasts and exclusively in the cytoplasm of myotubes. This differs slightly from murine muscle cells, in which ANXA1 is exclusively cytoplasmic in both muscle cell types [31]. As previously described for other cell lines [32], we observed that ANXA2 was localized exclusively in the cytoplasm of human myoblasts and myotubes (Figure 1B and Figure S2). Finally, endogenous or recombinantly expressed ANXA4 has been reported to be present in the cytoplasm and nucleus of various cell types [8,33]. In human skeletal muscle cells, we observed that ANXA4 was also present in both the cytoplasm and nucleus of myoblasts, but only in the cytoplasm of myotubes (Figure 1B and Figure S2).

We can conclude that ANXA1, ANXA2 and ANXA4 are expressed in human skeletal myoblasts and myotubes. They are all localized in the cytoplasm of myoblasts and myotubes and ANXA1 and ANXA4 are, in addition, found in the myoblast nucleus. Homogenous distribution of fluorescence within the cytoplasm suggested that these ANX are mainly cytosolic. ANXA3 may be absent from skeletal muscle cells, therefore making its involvement in membrane repair unlikely.

### 3.2. Trafficking of ANX after Sarcolemma Injury

Key membrane repair proteins, such as dysferlin [19], MG-53 [34] or ANX [3,14] are rapidly recruited to the membrane disruption site in damaged skeletal muscle cells. While most previous studies have been performed in mouse or fish, we investigated the trafficking of ANXA1, ANXA2 and ANXA4 fused to GFP in laser-damaged human LHCN myotubes. We have shown previously that LHCN myotubes are able to repair laser-induced sarcolemma damage within a minute timeframe [15,17].

**Figure 2:**
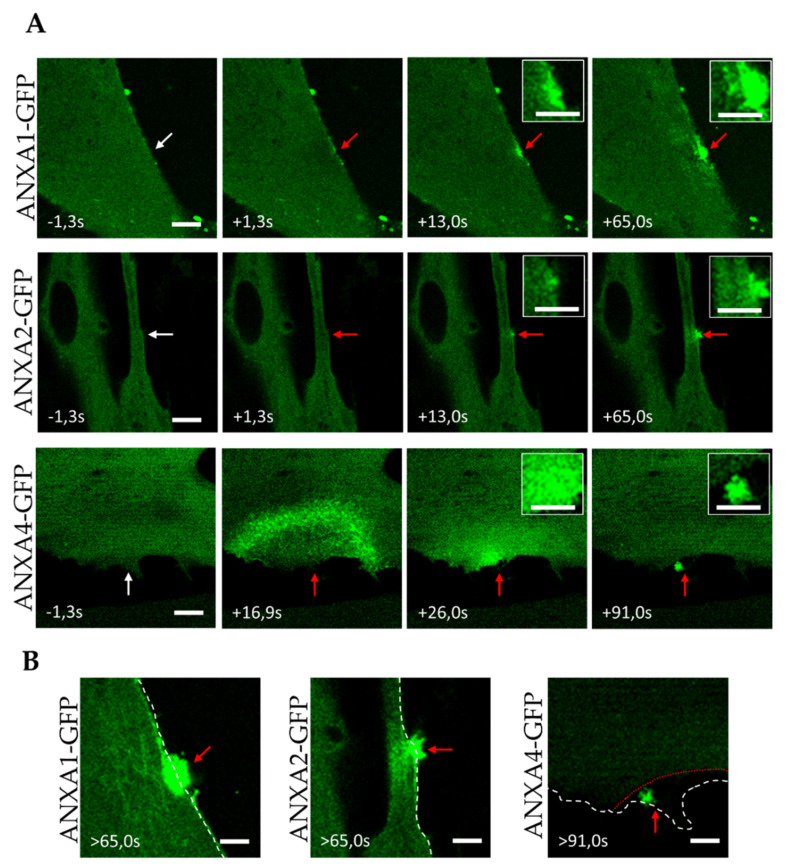
Recruitment of ANX to the site of membrane injury and formation of the cap subdomain in myotubes. (**A**) LHCN myotubes were transfected with the plasmid pA1-GFP, pA2-GFP or pA4-GFP and membrane damages were performed by laser ablation. White arrow, area before irradiation; red arrow, area after irradiation. Insets display magnified images of the membrane disruption site. Scale bar for images and insets, respectively: 10 µm and 5 µm. (**B**) Magnified images of the damaged myotubes presented in (**A**) more than 65 s after laser injury. The white and red dotted lines display the position of the sarcolemma before and after (if different) laser ablation, respectively. Scale bar: 5 µm.

We followed the recruitment of ANX-GFP to membranes (intracellular membranes or sarcolemma) after laser ablation by recording the local increase in fluorescence intensity, as previously reported [14,16,17]. We observed that ANXA1-GFP was immediately recruited to the damaged area (Figure 2A, Video S1). In contrast to ANXA6, which has been previously shown to interact with the plasma membrane [17], we observed that ANXA1 interacted mainly with cytoplasmic membranes (Figure 2A, +13.0 s). The punctiform distribution of ANXA1-GFP around the wounding site suggested interaction with intracellular vesicles or organelles (Figure 2A, +13.0 s and +65.0 s). Over time, ANXA1-GFP gradually accumulated at the damaged membrane site, forming finally a well-defined and dense structure, beyond the initial position of the sarcolemma (Figure 2A, +65.0 s and Figure 2B). This structure, revealed for the first time in damaged murine muscle fibers, has been called the “cap” subdomain [16]. The cap subdomain was initially defined as an organized protein scaffold, involving ANX and actin, that managed membrane resealing [16]. However, we have shown recently by TEM imaging that it likely corresponded to an accumulation of disorganized membrane materials at the surface of the repaired sarcolemma, in which coexisted proteins, including ANX [17].

ANXA2-GFP exhibited a trafficking similar to ANXA1-GFP, with a recruitment to the membrane disruption site through interactions mainly with cytoplasmic membranes (Figure 2A and Video S2). If a part of ANXA2-GFP accumulated into the cap subdomain about one minute after laser injury, a substantial part of ANXA2 remained instead localized deeply in the cytoplasm, as observed for ANXA1-GFP (Figure 2B).

**Figure 3:**
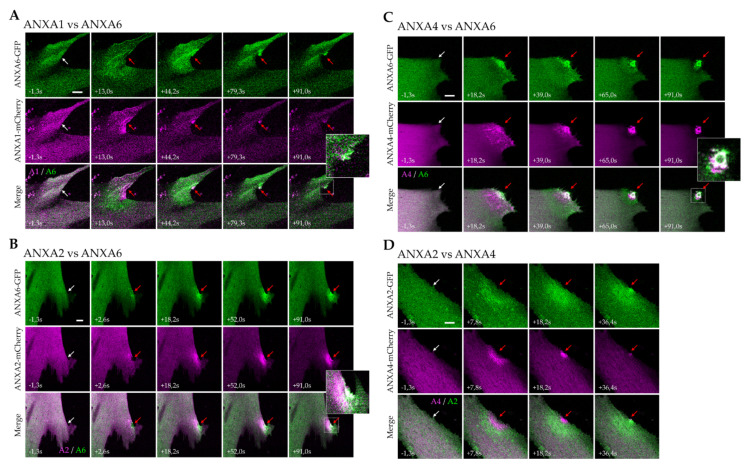
Comparative analysis of the recruitment of ANX to the site of membrane injury. (**A**) ANXA1 vs. ANXA6, (**B**) ANXA2 vs. ANXA6, (**C**) ANXA4 vs. ANXA6, and (**D**) ANXA2 vs. ANXA4. Myotubes were transfected with the plasmid pA1-mCherry, pA2-mCherry (or pA2-GFP for D), pA4-mCherry and pA6-GFP, as indicated. Membrane damage experiments were performed by laser ablation. White arrow, area before irradiation; red arrow, area after irradiation. Insets display magnified images of the membrane disruption site. Scale bar: 10 µm.

Finally, when ANXA4-GFP expressing myotubes were damaged by laser ablation, we observed a recruitment of ANXA4 that started far away (around 20 µm) from the site of the membrane damage and propagated in a wave-like fashion to the disruption site (Figure 2A and Video S3), where it finally concentrated exclusively in the cap subdomain (Figure 2A,B). As far as we know, the involvement of ANXA4 in the membrane repair of muscle cells has not yet been investigated. However, studies performed in cancer cells have shown that ANXA4 accumulated at the damaged area, where it may induce invagination of the membrane wound, in order to facilitate the constriction of the hole by ANXA6 [8]. The wave-like propagation of ANXA4, so far never observed for ANXA1, ANXA2 (Figure 2 and [14]), or ANXA5 [15], in muscle cells, is a property shared with ANXA6 (see Figure 3, Video S4–S6 and [17]). It is difficult to determine what mechanism drives the recruitment of ANXA4 to the sarcolemma disruption site. It should be kept in mind that the observation was made by a confocal microscope, which provided optical sectioning of the biological sample. The behavior of ANXA4, observed in Figure 2A, may be seen as a propagation along the inner leaflet of the sarcolemma, from the cytoplasm to the cap subdomain. In that case, ANXA4 could induce membrane remodeling that would promote membrane resealing, as proposed in cancer cells [8]. In addition, it has been reported that ANXA4 was able to aggregate membranes in the presence of Ca^2+^ [35], enabling ANXA4 to participate in membrane resealing by aggregating intracellular vesicles and/or sarcolemma.

### 3.3. Kinetics of ANX Recruitment to the Site of Membrane Injury

For further insight in the spatio-temporal trafficking of ANX in damaged human muscle cells, we performed the simultaneous observation of pairwise fluorescent protein-tagged ANX in damaged myotubes, by live imaging, using ANXA6 as a reference.

We first analyzed the behavior of ANXA1 and ANXA6, coupled respectively to mCherry and GFP, after membrane damage induced by laser ablation (Figure 3A, Video S4). We observed that ANXA1 was recruited earlier than ANXA6, to the site of the membrane injury (Figure 3A, +13.0 s). ANXA6 was recruited in a wave-like motion, to finally accumulate specifically in the cap subdomain, as previously shown [17]. However, ANXA1 accumulated gradually around the damaged area in the cytoplasm (Figure 3A, +13.0 s). It is striking to note that the ANXA1-mCherry fluorescence signal, accumulated at the membrane damage site, decreased after about 90 s, while that of ANXA6-GFP remained stable (Figure 3A, +91.0 s). Part of ANXA1 may therefore dissociate from the wounding site. ANXA1 has been described as a crucial component in the lipid patch, acting for the aggregation of intracellular vesicles [3], whereas ANXA6 may interact with the damaged sarcolemma, probably to induce the membrane remodeling responsible for membrane resealing [17]. Our data support this hypothesis, since ANXA1, interacting with intracellular vesicles and located inside the cell after membrane repair, may be released from the wounding site to return to the cytoplasm. Instead, ANXA6 interacting with the sarcolemma may face the extracellular space, where Ca^2+^ remains at mM concentration.

We then focused on ANXA2 and ANXA6 and observed that ANXA2 was recruited deeper in the cytoplasm and was present slightly later at the membrane disruption site, compared to ANXA6 (Figure 3B, +2.6 s and Video S5). This was particularly striking about one minute after the membrane damage, when ANXA6 was accumulated in the cap subdomain and a large part of ANXA2 remained concentrated inside the cell, just beneath the damaged area (Figure 3B, +91.0 s and Video S5). As described for ANXA1, this result supports the participation of ANXA2 in the formation of the lipid patch.

Regarding the recruitment of ANXA4 and ANXA6, we confirmed that both propagated to the disruption site in a wave-like fashion (Figure 3C, Video S6). ANXA6 was the first present at the disruption site (Figure 3C, +18.2 s), joined about 20 s later by ANXA4 (Figure 3C, +39.0 s). About 90 s after the sarcolemma injury, both ANX were exclusively present within the cap subdomain. If they colocalized in a central part of the cap subdomain, layers containing only ANXA4 or ANXA6 appeared on either side of this central part, which suggested the specific localization and putative specific function for each ANX. According to the model proposed in cancer cells by Boye et al. [8], ANXA4 induces a negative curvature to the damaged plasma membrane that will result in injury invagination, while ANXA6 allows for the constriction of the hole edges. In skeletal muscle cells, our observations showed that ANXA4 and ANXA6 were present outside the damaged myotube, in an interaction with a structure that seemed to protrude from the cell (Figure 3C, +91.0 s and Video S6). Whether the overall mode of action of ANXA4 (membrane curvature) and ANXA6 (membrane constriction) is similar in cancer and skeletal muscle cells or not, the mechanism would be slightly different, with an ANXA4-induced inward versus outward curvature, respectively.

Finally, since our results showed that ANXA2 and ANXA4 are recruited at the rupture site later than ANXA6, we wanted to determine the order of recruitment between both ANX. LHCN myotubes, co-expressing ANXA2-GFP and ANXA4-mCherry, were damaged by laser ablation for that purpose (Figure 3D and Video S7). We observed that ANXA2 was recruited deeper in the cytoplasm and was present at the rupture site after ANXA4 (Figure 3D, +7.8 s and +18.2 s). This different area of recruitment may correspond to different Ca^2+^ sensitivity between both ANX [32]. Less sensitive to Ca^2+^ than ANXA2, ANXA4 may bind to membranes closer to the site of the membrane injury. This experiment confirmed that ANXA4 was recruited in a wave-like motion, whereas ANXA2 accumulated progressively in the damaged area.

### 3.4. Localization of ANX at the Site of Membrane Injury Analyzed by CLEM

**Figure 4:**
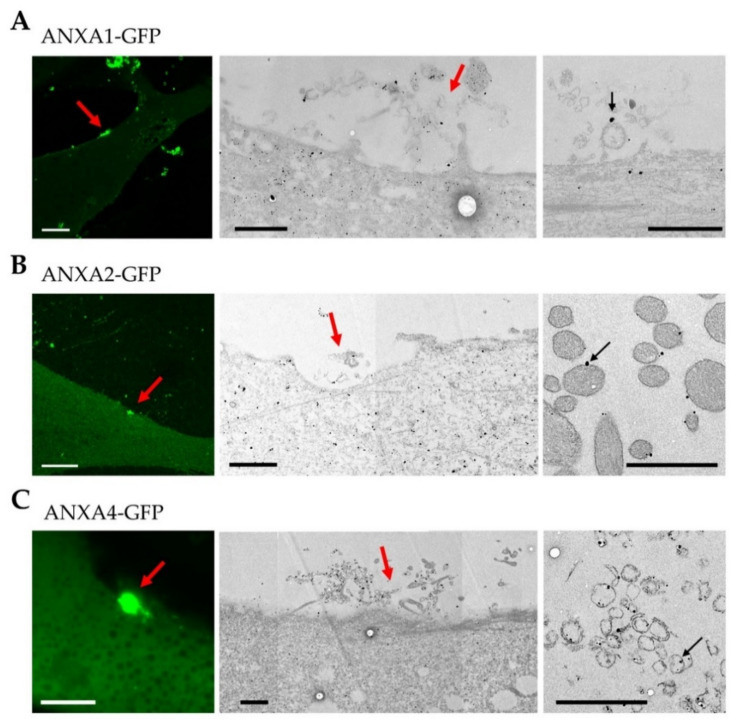
CLEM imaging of ANX in damaged LHCN myotubes. ANXA1-GFP (**A**), ANXA2-GFP (**B**), or ANXA4-GFP (**C**) expressing LHCN myotubes were damaged by laser ablation (red arrow) and immunostained for ANX using a secondary antibody coupled to gold nanoparticles (black particles). Fluorescence images obtained about 90 s after laser ablation are presented (left-hand image) together with TEM images (middle and right-hand images). Respective middle and right-hand images have been collected from different sections. Right-hand images show ANX (black particles) interacting with circular lipid structures (black arrow). Scale bar for fluorescence images: 10 µm; for TEM: 1 µm.

To get further insight into the distribution of ANX at and around the membrane disruption site, ANX-GFP expressing myotubes were damaged by laser ablation, chemically fixed and immunostained for ANX, using a secondary antibody, coupled to gold nanoparticles that enabled imaging by TEM, as previously described [36] (Figure 4). It is notable that the fixation process may have taken several minutes, even if the chemical fixative was added just after laser ablation. TEM images, therefore, mostly displayed ANX distribution once the sarcolemma was resealed. We observed systematically, the presence of the cap subdomain, suggesting membrane resealing (Figure 4). We confirmed that the cap subdomain is composed by an accumulation of disorganized cellular materials on the surface of the myotube (Figure 4) [17]. However, we noted within these complex materials, the presence of ANX-containing lipid structures (Figure 4, right-hand images). Major differences were nevertheless observed regarding ANXA1 and ANXA2, or ANXA4 and ANXA6. ANXA1 and ANXA2 were present on the surface of dense vesicular structures, whose appearance suggested intracellular vesicles (Figure 4A,B). These intracellular vesicles may have been detached from the lipid patch after membrane resealing, or having diffused from the cell just before this process. ANXA4 (Figure 4C) and ANXA6 (Figure S3) were instead inside circular structures displaying a weak electron density, looking like membrane fragments. It is likely that these fragments are of sarcolemmal origin.

## 4. Discussion

In healthy human skeletal muscle cells, we have shown that ANXA1, ANXA2, ANXA4, ANXA5 [15] and ANXA6 [17] are present in both myoblasts and myotubes, while ANXA3 is undetectable. Therefore, it is unlikely that ANXA3 participates in sarcolemma repair. The five other ANX have all been shown to be involved in membrane repair in different cell types [6,7,12,13,16]. As we have previously described the implication of ANXA5 and ANXA6 in human sarcolemma repair, we focused here on ANXA1, ANXA2 and ANXA4.

### 4.1. Ca^2+^ Sensitivity as a Driver of the ANX Machinery

**Figure 5:**
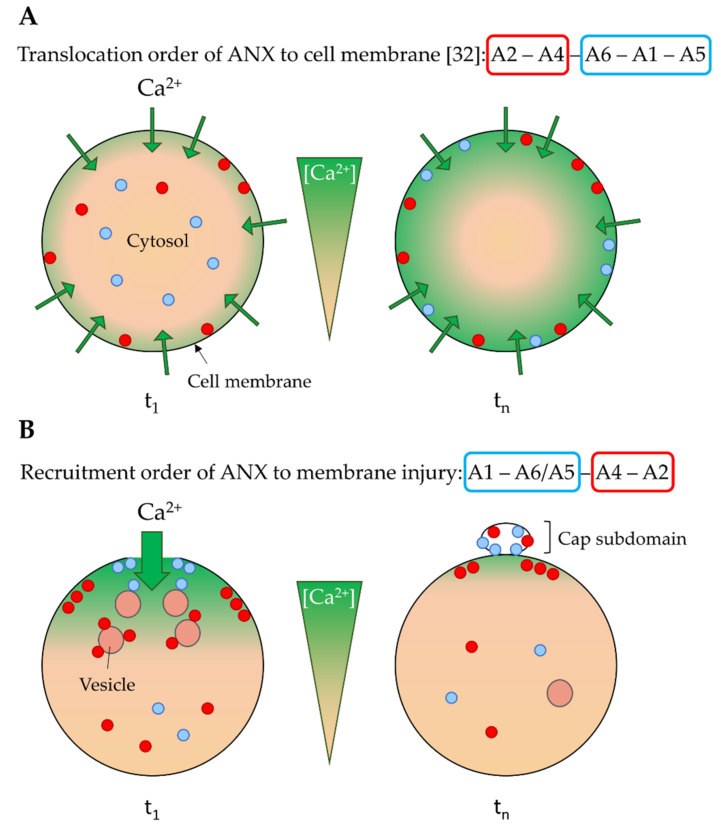
Model of cell membrane translocation and recruitment to the site of membrane injury of ANX according to their Ca^2+^ sensitivity. (**A**) As observed in Skrahina et al. [32], treatment with ionomycin, a Ca^2+^ ionophore, induces a gradual influx of Ca^2+^ in the whole cell (green arrows). At t_1_, first ANX to be translocated to the plasma membrane are therefore the most Ca^2+^ sensitive, such as ANXA2 and ANXA4 (red circles). At tn, high increase in the intracellular Ca^2+^ concentration causes the sequential translocation of ANX less sensitive to Ca^2+^ (blue circles). The order of translocation is as follows: ANXA2, ANXA4, ANXA6, ANXA1, and ANXA5. Data from [32]. (**B**) According to the current study, we propose that a local rupture in the plasma membrane causes a massive entry of Ca^2+^, creating a large gradient. At t1, the high Ca^2+^ concentration close to the disruption site induces the binding to plasma membrane of the lesser Ca^2+^-sensitive ANX, such as ANXA1, ANXA6 and ANXA5 (blue circles). Instead, high Ca^2+^-sensitive ANX, such as ANXA2 and ANXA4 (red circles), interact with membranes distant from the site of rupture. In human skeletal muscle cells, the order of recruitment to the site of membrane injury is the following: ANXA1, ANXA6/A5 [17], ANXA4, and ANXA2. Once membrane has been resealed (t_n_), ANX present in the cap subdomain, which is localized outside the cell where the Ca^2+^ concentration is high, remain in interaction with membranes. Inside the cell, most ANX, except eventually ANXA2, which is the most Ca^2+^-sensitive, are released from membranes when Ca^2+^ homeostasis is restored.

We show here that ANXA1, ANXA2 and ANXA4 are recruited to the site of the membrane injury of a damaged myotube within a minute, which is the time required for membrane resealing [15,17], suggesting these ANX may play a role in membrane repair. There are, nevertheless, differences in the kinetics of recruitment. We observed the presence of ANX at the disruption site, in the following chronological order: ANXA1, ANXA5/A6 [17], ANXA4, ANXA2. This is consistent with a previous study in human primary endothelial cells [10]. Surprisingly, this order is inversely correlated with ANX translocation to the plasma membrane, in cells treated with a Ca^2+^ ionophore [32]. It has been observed that the ionomycin treatment of murine neuroblastoma cells results in translocation to the cell membrane of ANX, in the following order: ANXA2, ANXA4, ANXA6, ANXA1, ANXA5 [32] (Figure 5A). This discrepancy may result from different cellular Ca^2+^ gateways between both conditions, in relation to the Ca^2+^ sensitivity of ANX. Ionomycin treatment and membrane injury lead to a global progressive, versus local massive increase, of intracellular Ca^2+^ concentration, respectively. After the addition of ionomycin (Figure 5A, t1), the Ca^2+^ concentration gradually increases, leading first to the translocation of the more Ca^2+^-sensitive ANX, i.e., ANXA2 and ANXA4. For an extended time, all ANX, even weakly sensitive to Ca^2+^, are in interaction with the cell membrane (Figure 5A, tn). Translocation to the cell membrane is, therefore, directly correlated to the Ca^2+^ sensitivity of ANX. In the case of mechanical damage of the plasma membrane, the rupture induces a massive entry of Ca^2+^, creating a large gradient, with a high Ca^2+^ concentration near the wounding site (Figure 5B, t1). While the interaction of Ca^2+^ weakly sensitive ANX with membranes (sarcolemma or intracellular vesicles) is privileged close to this site, Ca^2+^ highly sensitive ANX are “trapped” by association with membranes distant from the site of the membrane injury (Figure 5B, t1). Their recruitment to the wounding site is, therefore, slowed down and requires more time. Once the cell membrane has been resealed, most ANX are present in the cap subdomain, which is localized outside the cell, where the Ca^2+^ concentration remains high (Figure 5B, tn). Inside, ANX are released from membranes when Ca^2+^ homeostasis is restored, except eventually for ANXA2, which is the most Ca^2+^-sensitive. ANXA2 has been shown to be associated with membranes in the intracellular area beneath the wounding site, where it could reshape the cortical cytoskeleton for cell membrane renewal [7].

### 4.2. Implication of ANX in Sarcolemma Repair

**Figure 6:**
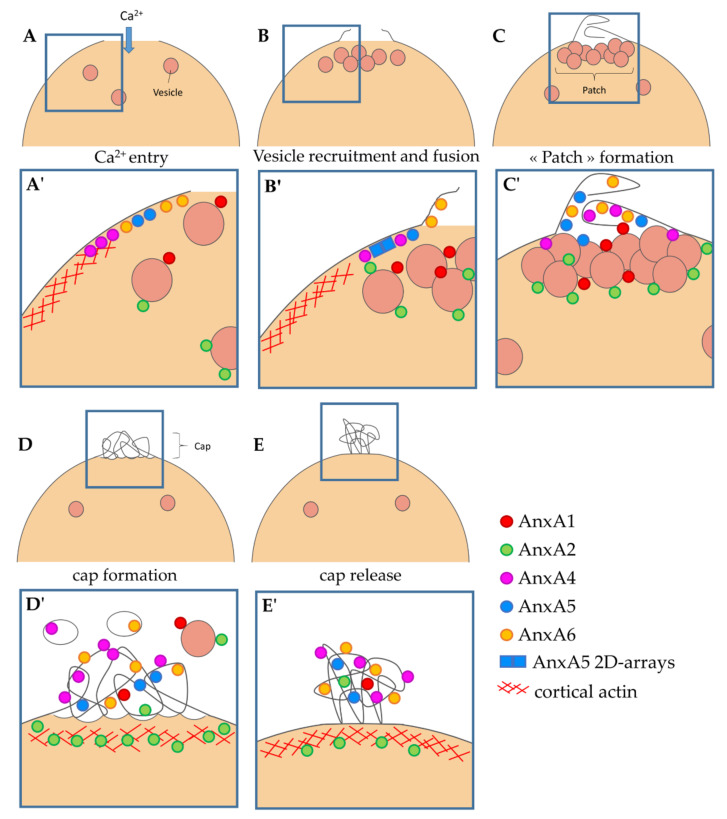
Model of cell membrane repair in human skeletal muscle cells. (**A**,**A’**) Entry of Ca^2+^ induces the recruitment of ANX to injured sarcolemma, notably ANXA5 [15], ANXA6 [17] and ANXA4. It induces also the recruitment of ANXA1/A2-bearing intracellular vesicles. (**B**,**B’**) Membrane tension is reduced by depolymerization of actin and exocytosis of lysosomes [37]. Increase in sarcolemma surface leads to excess membrane at the disruption site, on which ANXA6 is associated [17]. ANXA5 forms 2D arrays that strengthen the sarcolemma and limit the expansion of the tear [15]. Intracellular vesicles are recruited to the disruption site. (**C**,**C’**) Aggregation of intracellular vesicles forms the lipid patch that plugs the rupture. ANXA4 and ANXA6 induce folding of the extensions of sarcolemma in order to form a tight structure. (**D**,**D’**) Accumulation of ANX leads to folding and curvature of membranes [38] and the formation of the cap subdomain [16,17]. ANXA2 may promote repolymerization of cortical actin [7]. At this time, some vestiges of the lipid patch (rich in ANXA1 and ANXA2) and sarcolemma fragments (rich in ANXA4 and ANXA6) are present in the extracellular milieu. (**E**,**E’**) Integrity of sarcolemma is restored by membrane reconstitution and elimination of the cap subdomain by macrophages (not represented) [39]. Adapted from [17].

CLEM analysis, conjugating ANX-FP tracking and immuno-TEM, provides important clues for deciphering the specific functions of ANX in membrane repair. We have recently proposed a model of membrane repair in human skeletal muscle cells [17] that we are now able to complete (Figure 6).

An influx of Ca^2+^, subsequently to the membrane injury, induces the binding of ANX to surrounding membranes. The strong Ca^2+^ gradient leads to the recruitment of ANX less sensitive to Ca^2+^, to (plasma or vesicular) membranes close to the rupture site, and the recruitment of the most Ca^2+^-sensitive to ANX, to more distant membranes. ANXA1 and ANXA6 are, thus, more prone to interact at a closer distance to the wounding site when ANXA2 and ANXA4 are “trapped” far away from this site (Figure 6A,A’).

The recruitment of ANXA4 and ANXA6 to the site of the membrane injury occurs according to a wave-type propagation mode and ends in a structure, defined by McNally and collaborators, as the cap subdomain [16] (Figure 6C,C’). The molecular driving force responsible for this recruitment remains to be identified. On the other hand, ANXA1 and ANXA2 exhibit progressive recruitment to the site of the membrane injury, with a localization that remains cytoplasmic throughout the process (Figure 6B,B’). Once the injured membrane is resealed, a large part of ANXA1 returns to the cytosol, probably in relation to its low sensitivity to Ca^2+^ (Figure 6D,D’). At this time, we observed the presence of ANXA1 and ANXA2 on the surface of dense lipid vesicles, and ANXA4 and ANXA6 inside empty circular membrane fragments (Figure 6D,D’), which supports the hypothesis that ANXA1 / A2 and ANXA4 / A6 interact mainly with intracellular vesicles and the sarcolemma during membrane resealing, respectively. ANXA2 remains concentrated beneath the rupture point for an extended time, where it could act to reshape the cortical actin cytoskeleton, as proposed in cancer cells [7]. The final step consists, most likely, in the elimination of the cap subdomain, which is transformed as a tight structure, thanks to membrane remodeling by ANX (Figure 6E,E’). This process of elimination may require helping cells, such as macrophages [36].

## Data Availability

The authors declare that all data supporting the findings of this study are available within the article and its supplementary Information.

## Supplementary Materials

The following are available online at https://www.mdpi.com/article/10.3390/membranes12020153/s1, Figure S1. Expression of ANX in human myoblasts and myotubes. Figure S2. Subcellular distribution of ANX in human myoblasts and myotubes. Figure S3. Correlative imaging of ANXA6 in damaged LHCN myotubes. Supplementary videos: Recruitment of ANXA1-GFP (Video S1), ANXA2-GFP (Video S2), ANXA4-GFP (Video S3), ANXA1-mCherry and ANXA6-GFP (Video S4), ANXA2-mCherry and ANXA6-GFP (Video S5), ANXA4-mCherry and ANXA6-GFP (Video S6), ANXA4-mCherry and ANXA2-GFP (Video S7).
