# Supplementary material for: Trafficking of Annexins during Membrane Repair in Human Skeletal Muscle Cells
Source: Membranes (Basel). 2022 Jan 26;12(2):153. doi: 10.3390/membranes12020153 (PMC8877144; doi:10.3390/membranes12020153)
Supplement: Supplementary file 1 [file membranes-12-00153-s001.zip › Supplemental_Material_Membranes_Bouter.pdf]

membranes

Article

Trafficking of annexins during membrane repair in human skeletal muscle cells

Coralie Croissant, Céline Gounou, Flora Bouvet, Sisareuth Tan and Anthony Bouter *

Institute of Chemistry and Biology of Membranes and Nano-objects, UMR 5248, CNRS, University of Bordeaux, IPB, F-33600 Pessac, France.
* Correspondence: anthony.bouter@u-bordeaux.fr; Tel.: +33 540006860

SUPPLEMENTARY MATERIALS

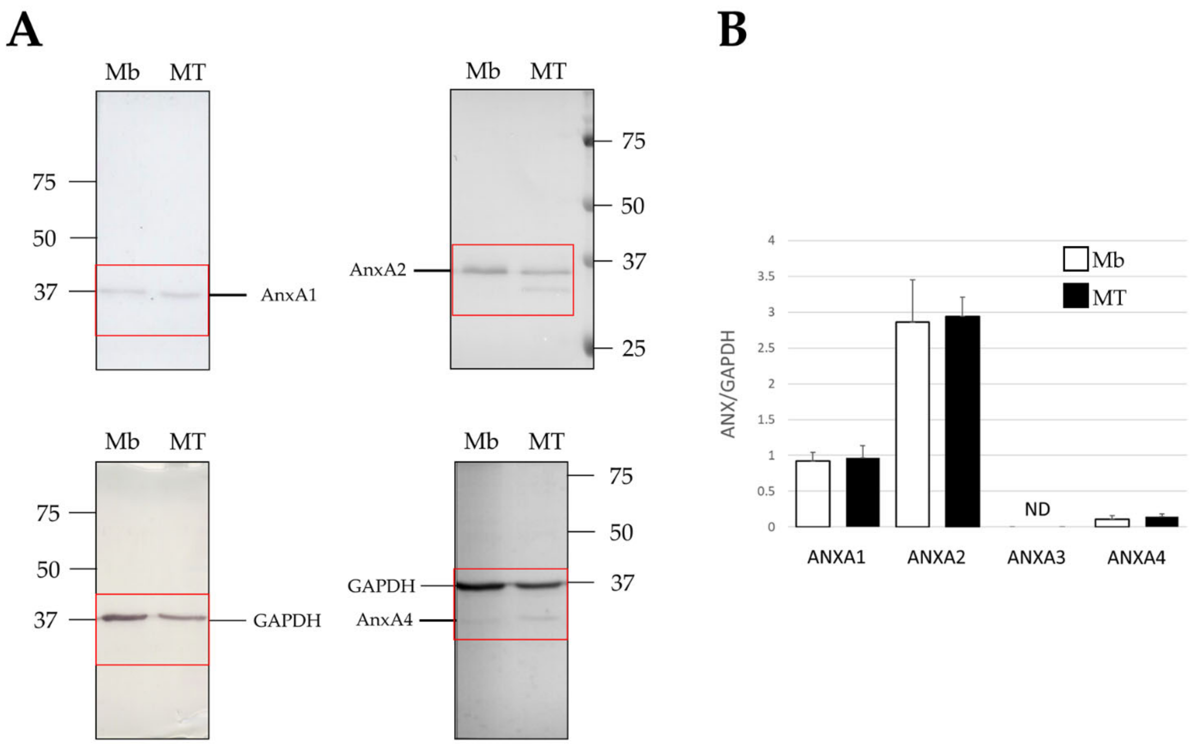

Figure S1. Expression of ANX in human myoblasts and myotubes. **(A)** Western blot experiments were performed as described in Figure 1A. GAPDH was used as loading control except for the detection of ANXA1 and A2, which present a similar molecular weight. In this case, a second membrane, loaded with strictly identical samples, was analyzed to verify that similar amount (10 µg) of protein extracts was used. Immunodetection of each ANX gave a unique band (two for ANXA2 in myotubes) at the expected apparent molecular weight. These results indicated the absence of cross-reactivity between the different antibodies used. Red boxes denote the regions of the original blots that are presented in the Figure 1A of the manuscript. **(B)** ImageJ software (Gels plugging) was used to measure the relative intensity of protein bands. Histograms represent mean (± S.D.) of ANX/GAPDH ratio calculated from at least three independent experiments. A Wilcoxon test was performed to identify putative statistical difference ($p<0.05$) between values obtained for myoblasts (Mb) and myotubes (MT). ND: Not detected.

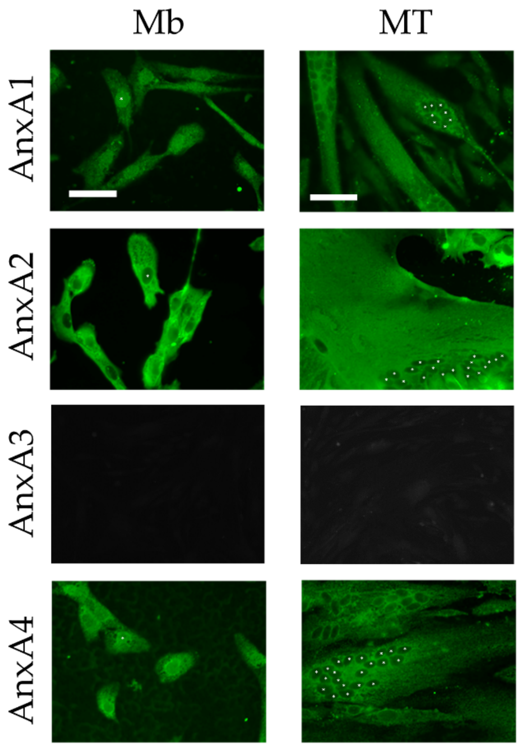

Figure S2. Subcellular distribution of ANX in human myoblasts and myotubes. Subcellular localization of endogenous ANXA1 to A4 (green) in LHCN myoblasts and myotubes by immunocytofluorescence. White asterisks indicate nuclei in one myoblast and one myotube per image. Scale bars: 50 µm.

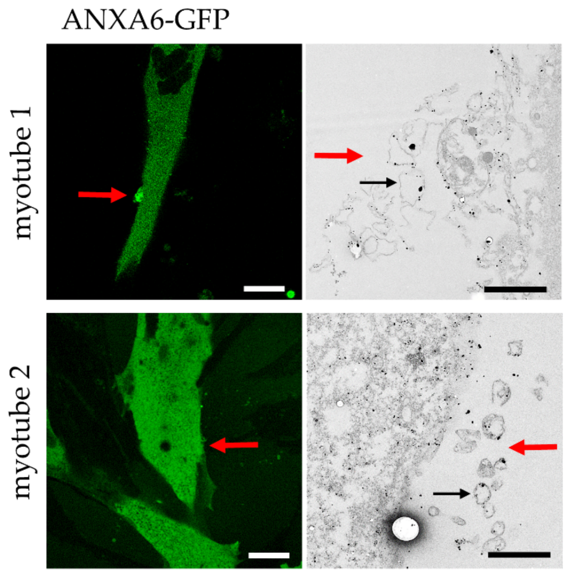

Figure S3. Correlative imaging of ANXA6 in damaged LHCN myotubes. Two different ANXA6-GFP expressing LHCN myotubes were damaged by laser ablation (red arrow) and immunostained for ANXA6 using a secondary antibody coupled to gold nanoparticles. Fluorescence image obtained about 90 s after laser ablation is presented (left-hand image) together with TEM images (right-hand images). The right-hand images show ANXA6 (black particles) inside circular structures (black arrow). Scale bar for fluorescence images: 10 µm; for TEM: 1 µm.
